# Many Paths to the Same Goal: Balancing Exploration and Exploitation during Probabilistic Route Planning

**DOI:** 10.1523/ENEURO.0536-19.2020

**Published:** 2020-06-10

**Authors:** Brian J. Jackson, Gusti Lulu Fatima, Sujean Oh, David H. Gire

**Affiliations:** Department of Psychology, University of Washington, Seattle, WA 98195

**Keywords:** Bayesian, foraging, navigation

## Abstract

During self-guided behaviors, animals identify constraints of the problems they face and adaptively employ appropriate strategies (Marsh, 2002). In the case of foraging, animals must balance sensory-guided exploration of an environment with memory-guided exploitation of known resource locations. Here, we show that animals adaptively shift cognitive resources between sensory and memory systems during foraging to optimize route planning under uncertainty. We demonstrate this using a new, laboratory-based discovery method to define the strategies used to solve a difficult route optimization scenario, the probabilistic “traveling salesman” problem (Raman and Gill, 2017; Fuentes et al., 2018; Mukherjee et al., 2019). Using this system, we precisely manipulated the strength of prior information as well as the complexity of the problem. We find that rats are capable of efficiently solving this route-planning problem, even under conditions with unreliable prior information and a large space of possible solutions. Through analysis of animals’ trajectories, we show that they shift the balance between exploiting known locations and searching for new locations of rewards based on the predictability of reward locations. When compared with a Bayesian search, we found that animal performance is consistent with an approach that adaptively allocates cognitive resources between sensory processing and memory, enhancing sensory acuity and reducing memory load under conditions in which prior information is unreliable. Our findings establish new approaches to understand neural substrates of natural behavior as well as the rational development of biologically inspired approaches for complex real-world optimization.

## Significance Statement

Animals display remarkable problem-solving abilities across a variety of complex situations. Here, we used a large, computer-controlled foraging field with precisely controlled probabilities of food resources in either repeated or random locations to test how rats determine which strategies to use to solve an extremely complicated route planning problem. We found that rats balanced exploration for novel locations of food with exploitation of known food locations to solve this problem, with the balance between exploratory and exploitative strategies governed by the amount of information available regarding resource location. Our results show how animals balance sensory input with learned information to solve complex, real-world route planning problems.

## Introduction

Animals balance the ability to flexibly interact with their environment with the need to reserve energy while foraging. Foraging in natural environments can be particularly difficult due to the sparse and unreliable nature of sensory cues emanating from food sources. This is especially true when animals need to travel between multiple locations and it is unknown whether food will be present at these locations. Under conditions of high uncertainty, it may be beneficial to rely on sensory information during foraging and use a more exploratory approach, when the increased cognitive demand of this strategy is offset by the need to flexibly interact with the environment. Conversely, using a memory-based strategy to exploit known resource locations allows for the quick establishment of efficient stereotyped routes, yet result in behaviors that are not readily adaptable to changing contingencies in the environment. It is therefore important for animals to maintain cognitive flexibility while foraging in their natural environment to execute the most efficient behaviors required for food procurement (Dolan and Dayan, 2013). To this end, the ability to adaptively modify search strategy by using internal representations of the dynamic environment would serve to vastly increase the effectiveness of foraging bouts (Slotnick, 2001; Zhang and Manahan-Vaughan, 2015).

Animals must learn the constraints of their environment to determine how to optimize their foraging strategies, with the balance of exploration versus exploitation being vital in this context (Kramer and Weary, 1991; Auh and Menguc, 2005; Gupta et al., 2006; Mehlhorn et al., 2015). During exploration, animals sample from multiple food patches over the course of several foraging bouts. This allows them to construct an internal representation of different possible locations where they can find food, with the benefit being that their future foraging would be more resistant to reduced or noisy sensory cues. Exploitation of this information follows and relies on remembering bountiful patch locations so that animals have a framework to use for navigation. While benefits of exploitation include spending less energy traveling to locations where it is unknown whether food will be available, potential drawbacks would be that this strategy fails when resources have been exhausted or when resource locations change. Additional exploration after establishing resource location is thus most useful when new resource locations need to be discovered, such as when information regarding resource locations is found to be unreliable. Under the constraints of foraging in an unpredictable environment, it is more difficult to exploit reliable resource locations to reduce foraging costs and strategies should shift toward exploration.

The ability to rapidly solve complex problems, such as optimization of foraging strategies, is a defining feature of animal intelligence. Indeed, varieties of animals solve difficult optimization problems nearly instantaneously (Wall and Balda, 1977; Kenward et al., 2005; Drea and Carter, 2009; Zhang et al., 2015). However, it has been difficult to study route optimization during naturalistic foraging in a laboratory setting. Historically, many foraging tasks have been studied with apparatuses that do not explore the full behavioral repertoire of a natural forager. One issue is the difficulty of providing alternative possible paths for the animals when they are restricted to a track, such as a figure-8 maze (Pedigo et al., 2006). In these simplified tasks, the space of available behaviors is limited to simple actions such as left and right turns. While other studies avoid these restrictions through the use of open field designs, these approaches necessarily reduce the precision and reproducibility of resource locations (Agarwal et al., 2014). We address these challenges by studying naturalistic foraging in a large, computer-controlled open field where food rewards can be precisely and reproducibly located anywhere in the environment.

Using our computer-controlled open field design, we investigated the strategies rats use to solve a notoriously difficult optimization scenario, the probabilistic traveling salesman problem. In this problem, an agent must establish the most efficient (i.e., shortest) route between a finite number of locations, and each location has a certain probability of containing pellets (Leipälä, 1978; Percus and Martin, 1999). We observed rats’ ability to follow efficient acquisition sequences and measured how well animal performance correlated with memory-guided exploitative strategies or sensory-guided exploratory strategies as a function of the predictability of the pellet distributions on which animals were trained. These precise behavioral experiments suggest animals adaptively shift their reliance on sensory information in response to the reliability of the foraging environment.

## Materials and Methods

### Subjects

The experiments in this study were performed on 12 male Long–Evans rats, purchased from Charles River Labs and housed individually. All animals were maintained on a 12/12 h dark/light reverse schedule (lights off at 7 A.M.) with *ad libitum* access to water. After a week-long habituation to the animal housing facility, all animals were then sustained at 85% of their free-feeding body weight to maintain motivation. All tests were performed between 9 A.M. and 6 P.M., during the dark phase of the light cycle. Zeitgeber time (ZT; with ZT0 = lights on in the animal facility) of experiments was ZT14 to ZT23. To limit distal visual cues, all tests were performed under dim red light (∼660 nm). All experimental procedures were approved by the Institutional Animal Care and Use Committee at the University of Washington.

### Testing apparatus

The foraging arena was a large, fully enclosed open-field measuring 2.5 m in length, 1 m in width, and 1 m in height. The frame of the arena was constructed from T-slotted aluminum railings. The sides of the arena were constructed from 1.27-cm-thick clear acrylic, while the ceiling was 0.635 cm in thickness. The floor was a sheet of 0.0635-cm-thick opaque white acrylic. The ends of the arena were made from a wire mesh to allow for air to circulate throughout. A nest area where the animals would remain during the intertrial interval was attached to one end of the arena. The nest area was constructed from 1.27-cm-thick clear acrylic. Two synchronized cameras (The Imaging Source; DMK 23UP1300; frame rate 120 per second) were used to track the movement of the animals. An automated, custom-made pellet dispenser was used to bait the arena with 45-mg sucrose pellets (Bio-Serv). An Arduino Uno controlled the movement of the motors running the pellet dispenser, allowing movement in the *x*- and *y*-coordinate plane.

#### Estimation of odor cues

Odor cue dispersal in the arena was directly measured using an ethanol source and miniature ethanol sensors (Tariq et al., 2019) that were scanned in a grid across the arena. The maximal signal detected at each sensor location over 30 s was normalized. There was no flow imposed on the arena, which limited the dispersal of airborne odor cues.

### Behavioral paradigm

Before testing, all animals were habituated to the animal facility for one week. Animals then spent 2 d habituating to the attached waiting cage for ∼15 min at a time. In order to motivate animals to return to the waiting cage, sucrose pellets were placed in the cage every 2 min when a 1-s, 1000-Hz tone was played. They were then granted access to the test arena and were given 2–3 d to habituate to it. Animals were considered to have reached criterion when they were able to make three transitions between the waiting cage and test arena within 30 min.

Animals were placed into the waiting cage at the beginning of each testing session. Rats completed one session a day of three trials each. Before each trial, the automated pellet dispenser baited the arena with sucrose pellets organized into three clusters of approximately three pellets each. During foraging periods the dispenser was automatically lifted out of the arena so that the animals could not interact with it. Procedures differed only through the testing phase, when animals were assigned to forage within environments of high, medium, or low food location predictability. Animals trained on the environment with high food location predictability (*n* = 4) were overtrained on a single distribution of pellet locations that stayed consistent across trials and sessions. Animals foraging in the environment with low food location predictability (*n* = 4) were trained on unpredictable pellet distributions that changed across trials. All other animals (*n* = 4) were trained on a moderately predictable distribution of pellet locations that changed slightly over time. All rats were given a maximum of 30 min to eat all of the sucrose pellets during the session. The entire testing period lasted for 30–35 d with approximately five sessions a week.

### Experimental design and statistical analysis

No explicit power analysis was conducted to determine sample sizes. However, the number of animals used is consistent with experiments in the current literature. All analyses were conducted using MATLAB (MathWorks) on PC workstations running under the Windows 10 operating system. A custom LabView (National Instruments) program was used to collect the behavioral data, also on a PC running the Windows 10 operating system. Significant differences between groups were assessed with the Mann–Whitney *U* test followed by *p* value adjustment with false discovery rate when multiple comparisons were made. Error bars in figures report the standard error of the mean and significant differences are indicated with asterisks.

Predictability of pellet distributions was quantified using an across trial minimum distance metric, which, for each pellet in a given distribution, reports the minimum distance from that pellet to all pellets in the immediately previous distribution. Relative entropy (RE) is equivalent to Kullback–Leibler divergence and was calculated as: RE(P∥Q)=∫P(j)log(P(j))/Q(j)) for all points j in the current trial’s probability density function (P) and the probability density function calculated from all previous trials (Q). Before calculating the RE all distributions were convolved with a smoothing function, which was an averaging filter of width = 1 cm. RE is reported in bits.

For establishing optimal pellet acquisition sequences for each distribution, we used a genetic algorithm developed by Joseph Kirk: Fixed Start Open Traveling Salesman Problem - Genetic Algorithm. Briefly, this algorithm starts from a population of randomly generated paths that start at the entrance to the arena and travel to each pellet once. It then uses an iterative process wherein in each “generation” of solutions the fitness of every path in the population is evaluated; the objective function for fitness in this case is minimization of path length. The more fit (shorter) paths are selected, and each path’s sequence of pellet locations is modified (recombined with other paths or randomly changed, or “mutated”) to form a new generation. The new generation of candidate paths is then used in the next iteration of the algorithm. The algorithm can be terminated when either a maximum number of generations has happened or the path length reaches a small enough value.

Efficiency of foraging paths was calculated as fe=lo/la, where lo is the optimal path length, la is the animal’s path length, and fe is foraging efficiency.

### Bayesian search

For analyses conducted in Figure 5, we modeled rat behavior as a Bayesian search. Briefly, the search arena is divided into 2.8-cm squares resulting in a 40 × 80 grid of possible locations. This grid is then populated with the same pellet distributions that were used in the behavioral experiments. We start our analysis on day 10 of training, which provides an agent with up to the first 10 d of training data as a map of prior expectations regarding pellet locations (Fig. 5*A*). The expression for prior expectation of pellet location is given by:

$$pe(x,y)=\Sigma_{L}^{t-1}rw(x,y)/(t-L),$$

where *t* is the trial number, *rw* is the probability of a pellet being found at a given point, (*x*, *y*), over previous trials, and *pe* is the resulting prior expectation from the previous pellet locations. *L* is based on the length of memory being used and is defined as L=(t−md,1), with *md* being memory depth in trials, with md>=1. To enforce the nearest-neighbor search strategy used by rats, this map of prior expectations is discounted by linear distance from the agent, resulting in decreased likelihood to search first in areas that are located at large distances from the agent. This results in the following expression at a point, (*x*, *y*) within the grid of possible pellet locations:

m(x,y)=pe(x,y)∗((max(d)−d(x,y))/max(d)),

where *d* is the distance from the agent, and *m* is the memory-based map of prior expectations for pellet location adjusted by distance from the agent. The agent also uses sensory information that decays with distance to update their expectation of the possible pellet location,

$$s(x,y)=cr(x,y)*{\left((max(d)-d(x,y))/max(d)\right)}^{se},$$

where *s* is the sensory density function, and *cr* is a map with the current location of all pellets set to 1 and all other locations set to 0. The term *SE* is an exponent that determines the rate of decay of sensory information with distance. These two sources of information are weighted and then summed to result in a map that guides the agent’s next step in the search path,

p(x,y)=s(x,y)∗sw+m(x,y)∗(1−sw),

where *p* is the probability map, *s* is the sensory density function, and *m* is the memory-based map of prior expectations for pellet location. The term *sw* is the weight given to sensory information, {sw|0≤sw≤1}. The agent makes its next step along the vector to the maximum point of *p*. The agent is considered to have perfect target detection at their location, such that after the agent moves to a new location, if a pellet is at that location it is always detected and if no pellet is at that location the probability of a target at that site is updated to 0. To fit parameters for the Bayesian search, we used a three-dimensional coarse grid of values for *sw*, *SE*, and *md*. We found the best fit for each animal in this grid and report these results in Figure 5.

For reported measures in Figure 5*F*, sa=(1−(se/(SE))+sw)/2, where *sa* is sensory acuity, and *SE* is the set of values of *SE* across all best fits for 12 animals, while mi=(max(pr{md > 0})−mean(pr{md < 3}))/(max(pr{md > 0})−min(pr{md > 0})), where *mi* is long-term memory usage, and *pr* is the correlation of the agent’s performance with the animal’s performance using *md* set to the indicated range of values.

### Software accessibility

All software developed for analysis and generation of figures is available at the Gire Lab website and at GitHub.

## Results

### Route planning revealed through controlling predictability of reward locations

**Figure 1.**
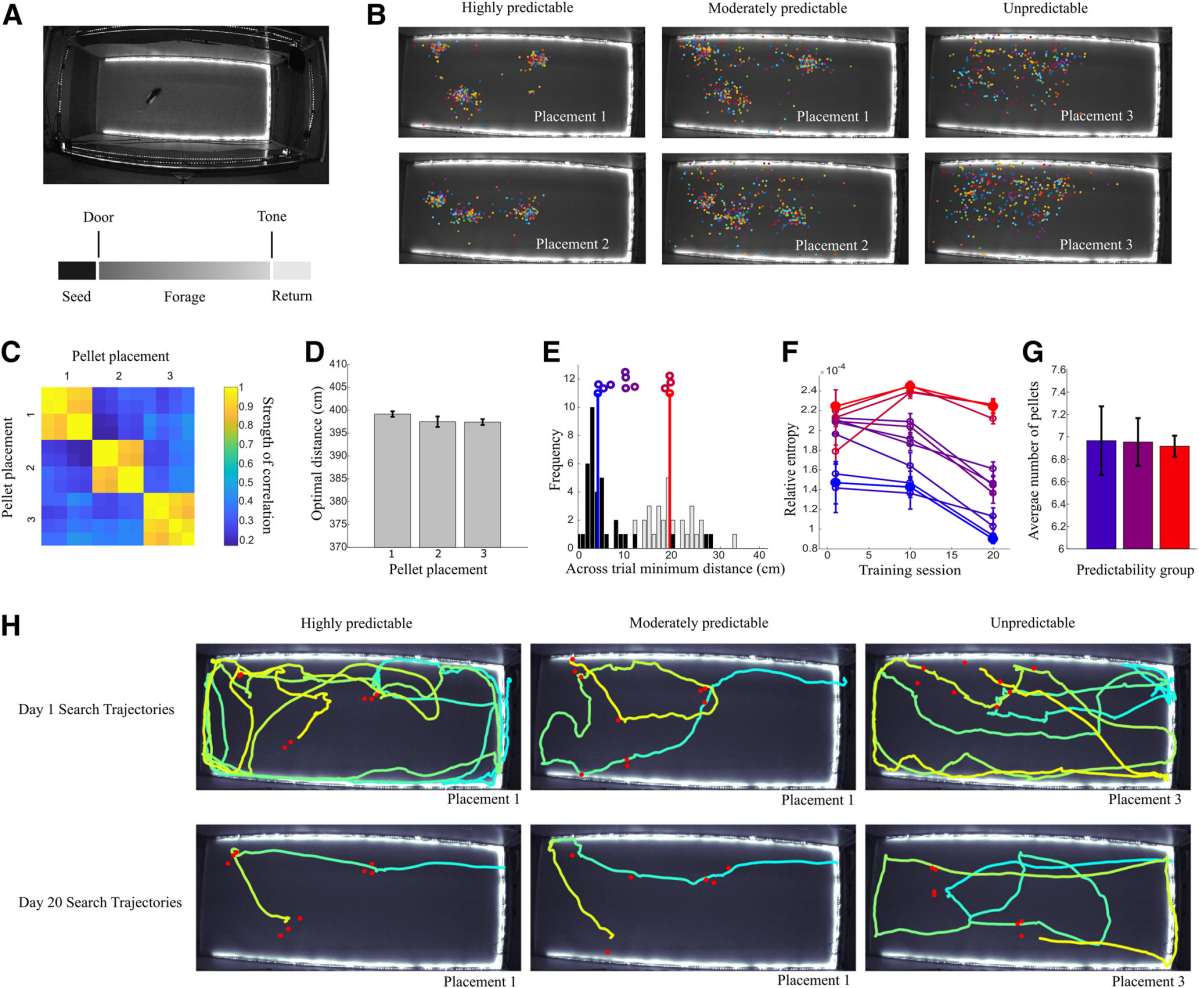
A computer-controlled probabilistic traveling salesman task enables direct tests of behavioral strategies under uncertainty. ***A***, top, A large, automated arena with a rat shown for scale. Bottom, The temporal structure of a typical trial. ***B***, Rats forage for pellets in highly predictable (left), moderately predictable (center), and actively randomized (right) pellet placements. Placements are shown across all trials (20 d, 3 trials per day). ***C***, The automated system allows for reproducible pellet placement across animals. From the top to bottom of the matrix correlation coefficients are shown for two different predictable distributions and the single unpredictable distribution. ***D***, Pellet distributions from each placement shown in panels ***B***, ***C*** have equivalent optimal path lengths. Error bars are standard error of the mean. ***E***, Example histograms are shown for the most predictable (black) and least predictable (gray) distributions that were tested. Vertical colored lines show the mean for the predictable (blue) and unpredictable (red) distributions. The distributions for all animals are plotted as colored circles, with color corresponding to across trial minimum distance. ***F***, RE for each predictability grouping (high, blue; medium, purple; randomized, red) across sessions of training. Higher values indicate higher entropy. ***G***, Average number of pellets per trial for each predictability level. ***H***, Examples of routes taken by rats on the first trial of the first day (top panels) and after 20 d of training (bottom panels). Color shifts from cyan to yellow as each animal’s trajectory progresses.

**Figure 2.**
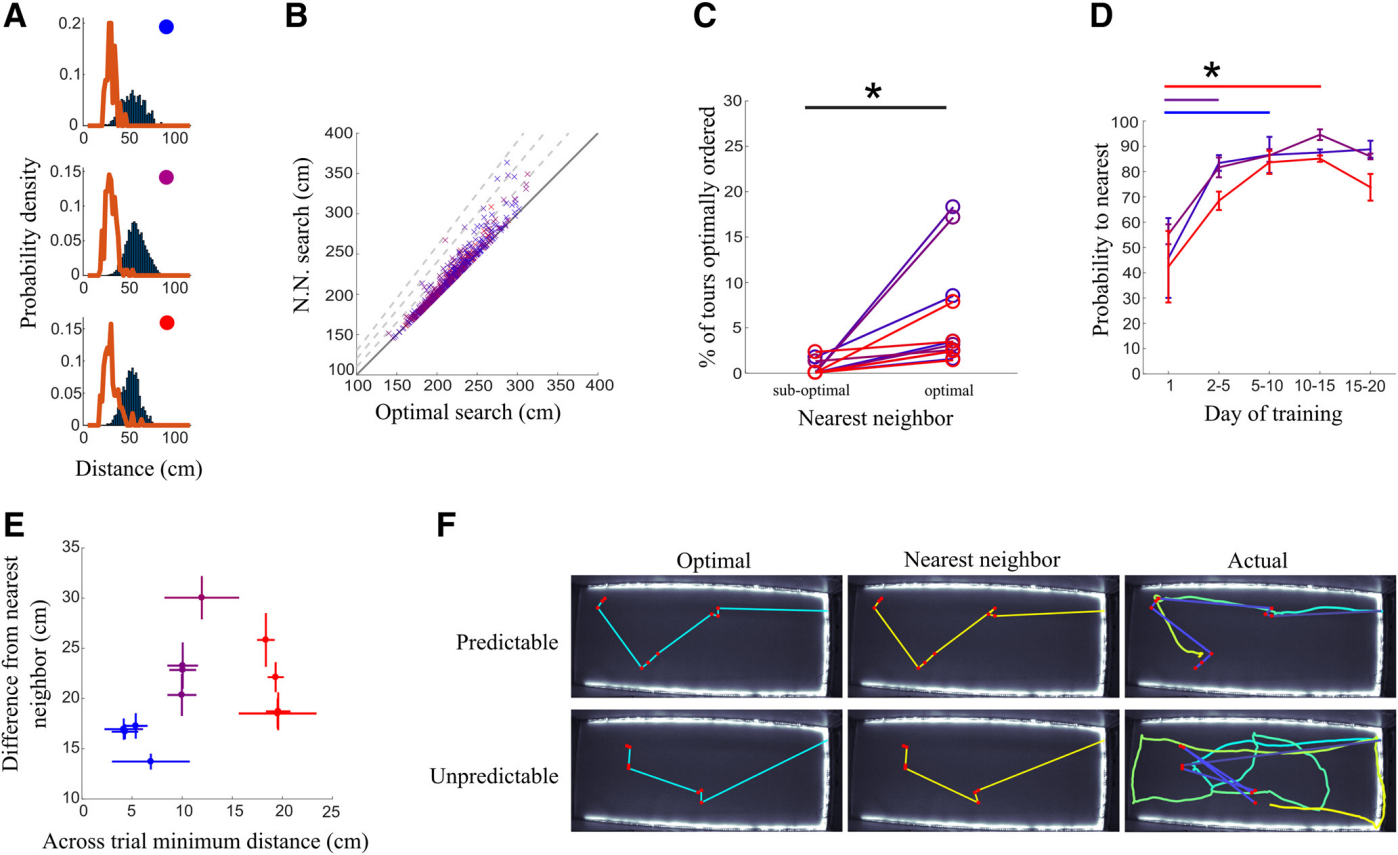
Search performance approaches a nearest neighbor heuristic after experience with reward locations. ***A***, Average distance per pellet. Rats acquire pellets in a sequence that is extremely efficient (red lines) compared with a random sampling of all possible sequences (blue bars). Predictability decreases from top to bottom. ***B***, Performance of a nearest neighbor strategy on all distributions tested in this study when compared with the optimal path length. Dashed lines represent 10%, 20%, and 30% above optimal. ***C***, Animal performance on trials in which a nearest neighbor search is optimal versus trials in which a nearest neighbor search is suboptimal. Asterisk indicates significant difference between the groups. ***D***, The probability that rats in each predictability group acquire the nearest pellet during search increases during training for all groups. The initial training epoch at which rats showed significant improvement from the first day is indicated by color-matched horizontal lines and asterisks. ***E***, Scatter plot showing the relation between predictability of distribution (*x*-axis) and difference between animal acquisition sequence and nearest neighbor sequence (*y*-axis) for all 12 animals. Error bars are standard error of the mean across trials for each animal. ***F***, Example of optimal and nearest neighbor pellet acquisition sequences and the actual sequences and trajectories taken by animals. For the right panels, color shifts from cyan to yellow as the animal’s trajectory progresses and from dark to light blue as the pellet acquisition sequence progresses.

We adapted the probabilistic traveling salesman problem for experimental investigation through the use of an automated system for precise, computer-controlled food pellet placement within a large foraging arena (Fig. 1*A*). We divided a cohort of 12 rats into three equal groups that foraged within environments of high, medium, and low food location predictability (Fig. 1*B*). Animals in each group were tested across precisely replicated pellet placements (Fig. 1*C*), and all placements used had equivalent optimal path lengths (Fig. 1*D*), as calculated through a genetic algorithm solution to the traveling salesman problem for each pellet placement (see Materials and Methods). We generated sequences of pellet locations over days to create distributions that were extremely well predicted by prior experience as well as distributions that were unable to be anticipated based on prior pellet locations. To generate pellet placements with controlled levels of predictability, we quantified the between trial minimum distance for each pellet of a given distribution and all pellets of the previous trial’s distribution and set this value to be low for the computer-generated set of locations used for predictable conditions and to be high for the unpredictable condition (Fig. 1*E*). The lower values for pellets in predictable distributions indicate that these pellets are in areas that are extremely close to where pellets were located on the previous trial, allowing animals to create an expectation over repeated searches. This is also demonstrated through a reduction of the RE (a measure of surprise) of newly-encountered pellet distributions following multiple days of training for animals in high and medium predictability conditions. Animals could not develop such an expectation under low levels of predictability and RE does not decrease with training for the unpredictable distribution (Fig. 1*F*). In all conditions, animals searched for an average of seven pellets, with the precise number on a given trial unknown to the animal (Fig. 1*G*). This results in typically 7!, or 5040 possible sequences of pellet acquisition, with most sequences being extremely suboptimal. Examples of trajectories taken by animals on the first and last days of training demonstrate changes in search trajectories with learning (Fig. 1*H*). After training, all animals favored a small subset of near-optimal acquisition sequences (Fig. 2*A*), consistent with findings in non-probabilistic optimization across a number of species (Blaser and Ginchansky, 2012). We found that a simple nearest neighbor heuristic (in which rats solve the task by traveling to the next nearest pellet) achieved strong performance on this task, often comparable to that of optimized routes (Fig. 2*B*). Indeed, we found that animals achieved optimal performance only when the optimal solution was the same as a nearest neighbor approximating solution (Fig. 2*C*), suggesting that the rats employed the nearest neighbor heuristic to solve the task. Rats foraging in predictable environments were capable of employing a nearest neighbor strategy earlier during training, although all animals, even those in unpredictable environments, did increase the use of nearest neighbor routes while foraging (Fig. 2*D*). However, animals in the highest predictability group were significantly more effective at ordering their search based on nearest neighbor relations of reward locations (error relative to a nearest neighbor search: 16.9 ± 0.5 cm for most predictable, 22.1 ± 2 cm for moderately predictable, and 20.8 ± 1.4 cm for least predictable, *n* = 4 animals per predictability group; Fig. 2*E*; for statistical tests used for all comparisons, see Materials and Methods). Examples of optimal, nearest neighbor, and animal sequences of pellet acquisition for animals in highly predictable and unpredictable environments are shown in Figure 2*F*.

### Predictable environments enable enhancement of search routes

**Figure 3.**
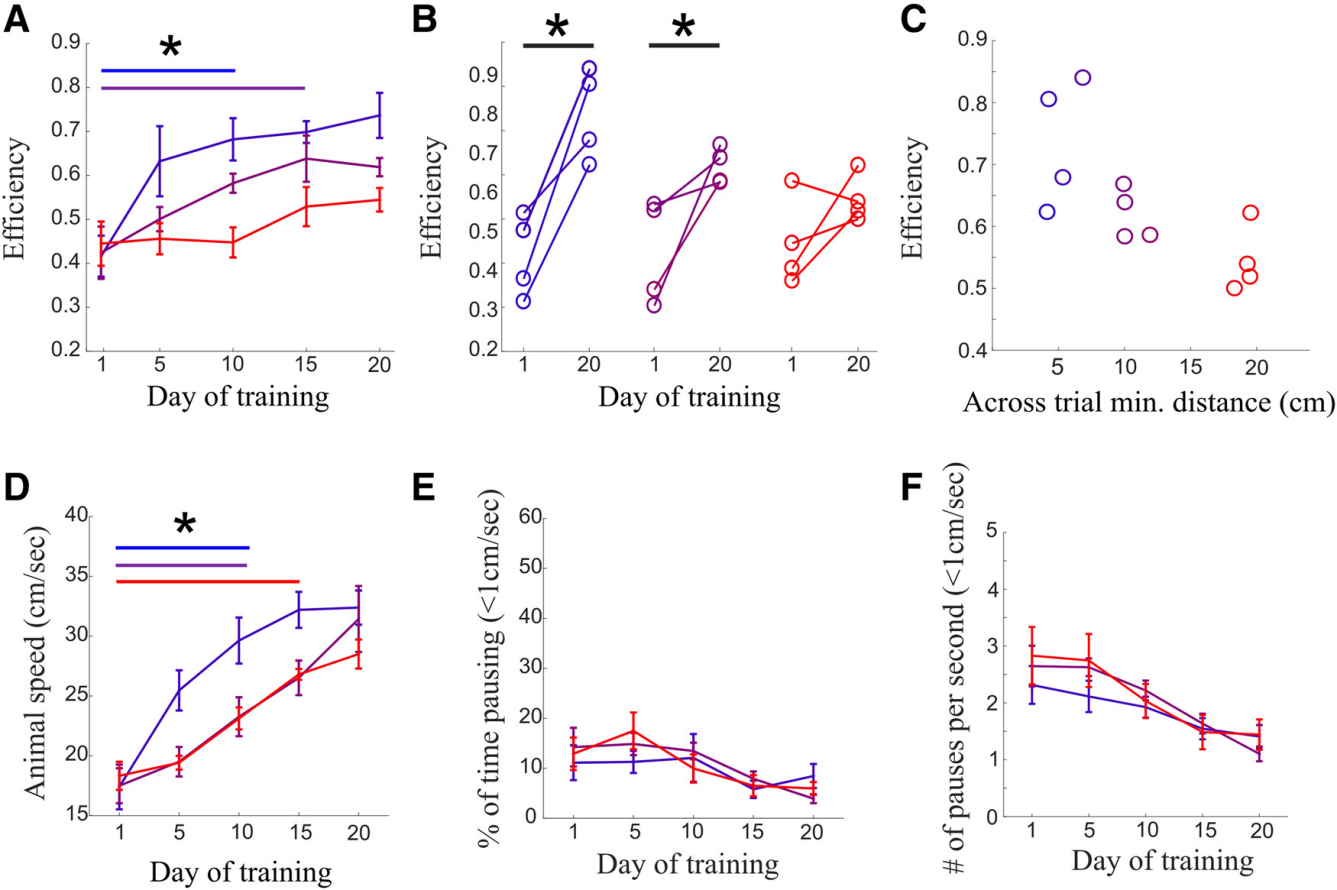
Predictability supports increased route efficiency. ***A***, Animals searching in predictable environments increase efficiency with training (for efficiency metric, see Materials and Methods). Efficiency was measured on paths to rewards that were located >40 cm away and were assessed on day 1 and then on blocks of 5 d until day 20. The first day of training that showed significant improvement in efficiency from the first day is indicated by color-matched horizontal lines and asterisks. ***B***, Animals in both predictable groups significantly increased the efficiency of their search routes on the last block of training when compared with the first day. Significant improvement is marked by a horizontal line and asterisk. ***C***, Efficiency of search routes measured on the last block of training (days 15–20) show a strong negative correlation to the unpredictability of the foraging environment, here measured as the cross trial minimum pellet distance (see Materials and Methods). ***D***, All animals increase speed during training. Average speed was taken without including pauses. Color matched horizontal bars indicate the first point at which speed was significantly greater than day 1 for each predictability condition. ***E***, Animals spend a small amount of time pausing during the task and this does not significantly change with training. ***F***, The number of pauses per route as a function of training.

In our task, which involves probabilistic presence of pellets, this nearest neighbor search can be implemented through two different strategies: in a sensory-guided strategy animals use cues (odor or vision) to navigate toward the nearest detected target; in a memory-guided strategy animals use prior information to navigate toward the nearest, most likely locations of pellets. We next investigated which of the two alternative strategies might guide a nearest neighbor search within each level of uncertainty. Over training, animals across all predictability levels significantly increased their probability to travel to the nearest pellet during search (Fig. 2*D*). However, the number of days of training taken for this to occur was dependent on the predictability of the pellet distribution (significant improvement on days 2–10 for highly and moderately predictable conditions, significant improvement not until days 10–15 for unpredictable conditions; *p* <0.05 compared with day 1, *n* = 4 for all groups; Fig. 2*D*). We found that animals searching in highly predictable environments were effective at enhancing the efficiency of their search across long distances (>40 cm) and learned to do this relatively early in training (days 5–10). Those in moderately predictable environments also learned to increase the efficiency of their search tours but required more training to do so (days 10–15), while those searching in unpredictable environments did not significantly increase the efficiency of their tours (Fig. 3*A*,*B*). As the unpredictable nature, or “surprise value” of the environment increased, the ability of animals to increase the efficiency of their search tours decreased (*R* = −0.72, *p* < 0.008, *n* = 12; Fig. 3*C*). These results suggest that based on the predictability of the environment rats employ two different strategies to find the next nearest pellet, one in which tours can be efficiently narrowed toward straight line paths and another in which paths between rewards are necessarily circuitous (Fig. 1*H*, lower panel for example tours after training).

In addition to supporting better-ordered search routes (Fig. 2*D–F*) and efficient paths to the nearest target from farther away (Fig. 3*A–C*), predictable distributions also enabled rats to enhance the speed of their travel between rewards. During training, the speed of the trajectories taken between pellets increased the most quickly for animals operating in the most predictable environments, although all animals eventually learned to decrease time between rewards by increasing speed (Fig. 3*D*). Time spent pausing (speed <1 cm/s) and number of pauses per second did not significantly change with training (Fig. 3*E*,*F*), suggesting consistent motivation to perform the task across all animals.

### Analyzing shifting weightings between sensory-dominated and memory-dominated strategies

**Figure 4.**
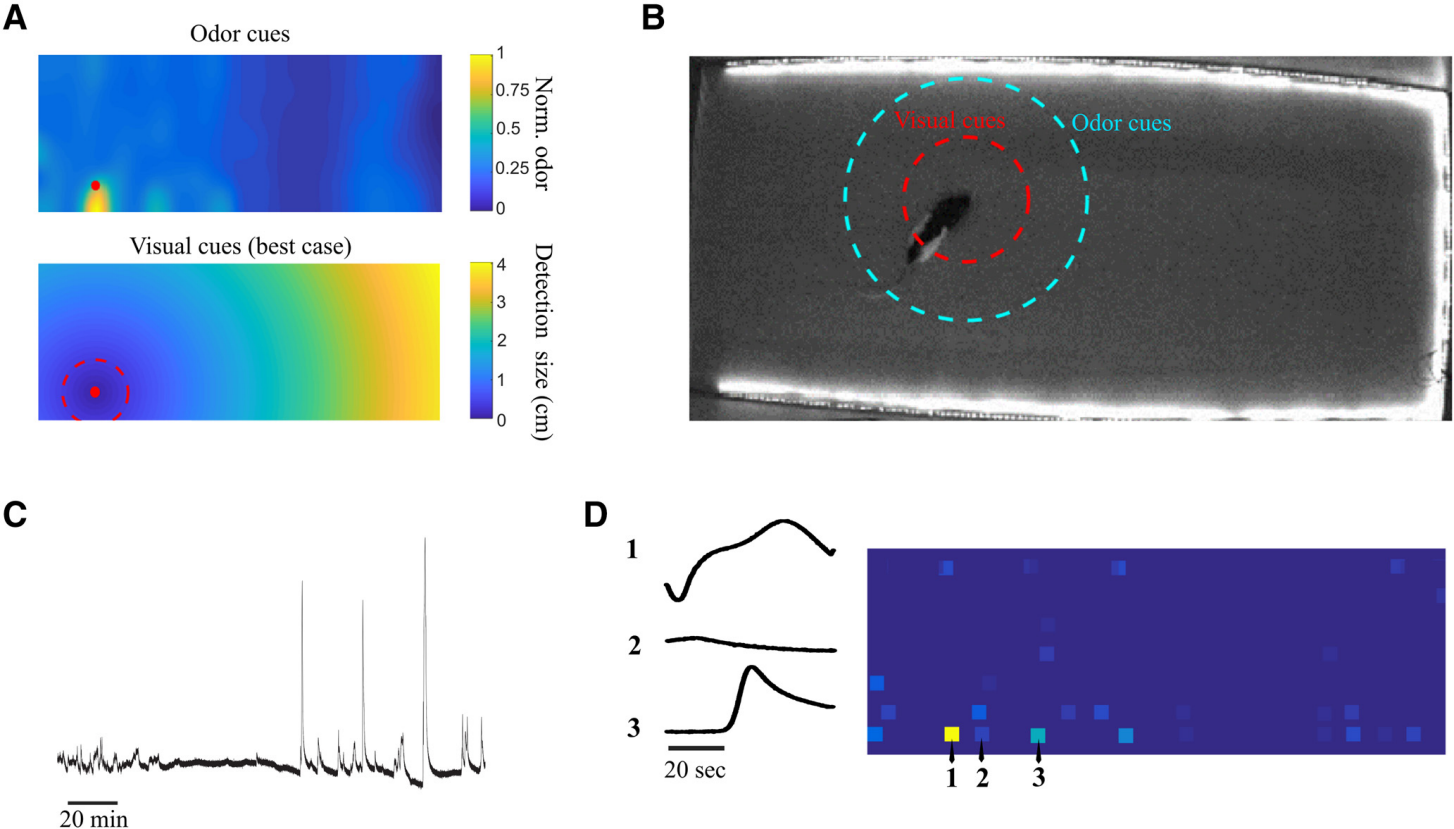
Sensory cues are local. ***A***, top, Experimentally determined spread of odor in the foraging arena (see Materials and Methods). Bottom, Calculated size of a pellet necessary for it to be visible for a foraging rat under bright, broad-spectrum lighting conditions with high contrast, based on reported values for rat visual acuity Prusky et al. (2000, 2002). The dashed red line indicates the actual size of the pellets used (and thus the distance for detection under ideal conditions). All experiments in the current study were done under dim red light using pellets matched in color to the arena floor, further limiting the range for visual detection. ***B***, Estimated best-case pellet detection distances for olfactory (cyan) and visual (red) sensory cues. Because of both the dim, red lighting conditions and the lightly odorized pellets, actual detection distances are likely to be much smaller. ***C***, The entire time course of odor for one mapping experiment (∼180 min) used to establish the distribution in panel ***A***. As the sensor is moved closer to the source (later in the experiment), odor fluctuations become much larger. ***D***, A grid of mean odor intensity values that were sampled during the experiment and convolved with a Gaussian function to create the estimated odor density function in panel ***A***. Odor sensor activation over time from the indicated locations (1, 2, and 3) is shown to the left of the grid.

**Figure 5.**
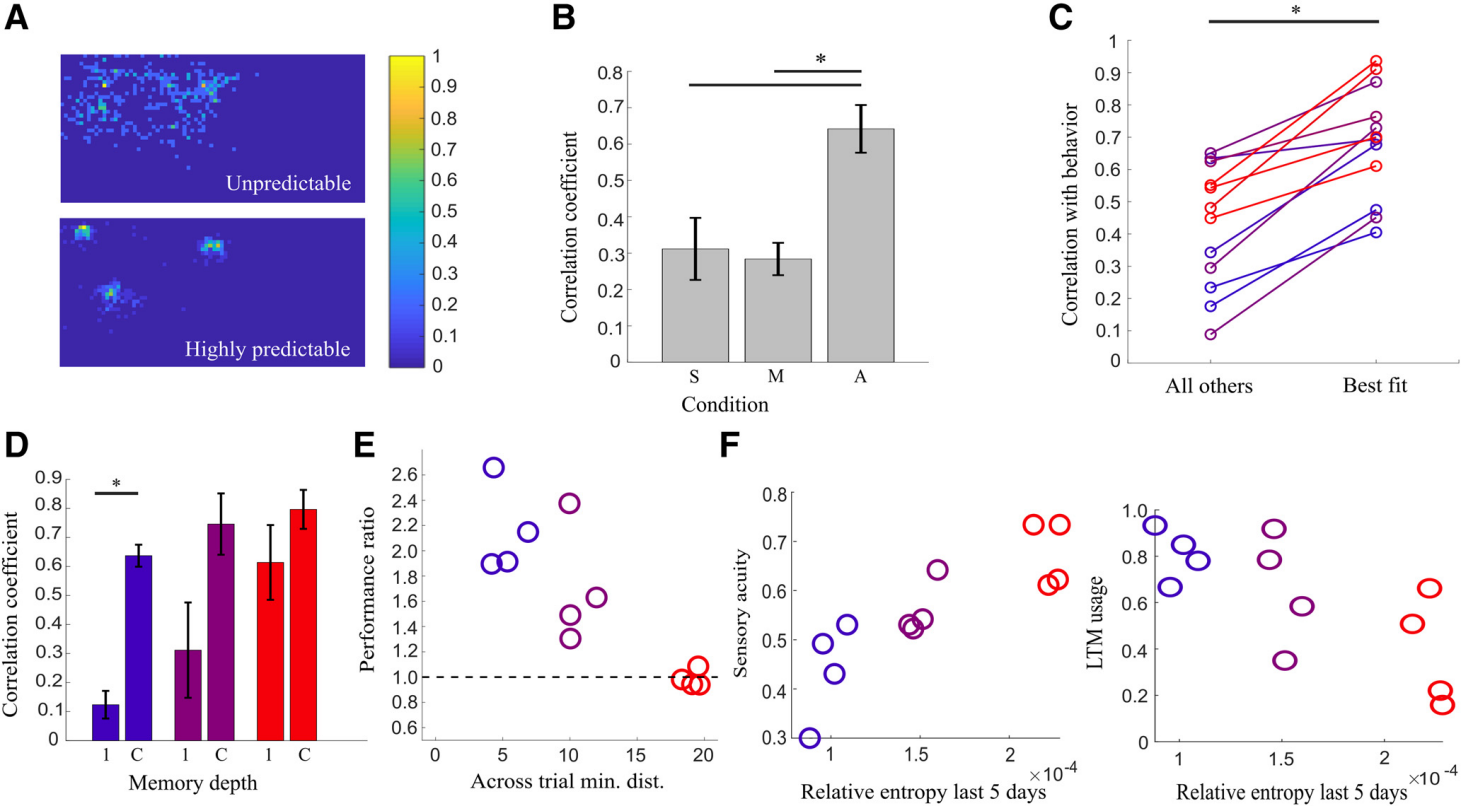
Modeling behavior as a Bayesian search with adaptive sensory acuity and memory depth explains performance under uncertainty. ***A***, Examples of prior distributions accumulated over all trials for one predictable and one unpredictable set of pellet locations. Distributions have been normalized to their largest value (color bar). ***B***, Correlation to animal performance of models with parameters emphasizing sensory (S) or memory (M) guidance or an adaptive model (A) individually fit to each animal. Horizontal lines indicate comparisons with significant differences between groups. ***C***, Correlation of agent’s search performance with animal behavior when using parameters fit to other animals (all others) or the best fit to that specific animal (best fit). The best fit is significantly better than the fits from other animals (*p* = 0.0043, *n* = 12; significance indicated by horizontal line and asterisk). ***D***, Correlation between animal behavior and a Bayesian search with either single trial memory (1) or best performance with cumulative memory (C). Significant difference is indicated by the horizontal line with an asterisk. ***E***, Performance ratio (path length with priors from different distributions/path length with correct prior) for all animals plotted as a function of the across trial minimum distance for the distributions presented to each animal (significant correlation: *R* = −0.85, *p* = 0.0004). A higher value for the performance ratio indicates longer path length with a shuffled prior. Agents searching with unpredictable distributions (red) show identical performance regardless of the prior used. ***F***, left, Sensory acuity based on the best fit search parameters versus RE based on the distributions that animals have experienced. Right, Long-term memory usage versus RE of pellet distributions encountered.

We next sought to more precisely quantify the role of sensory information and memory in the navigation strategies used by animals under varying levels of uncertainty. To perform this analysis we simulated animal behavior by developing an agent that searched through foraging space using multiple free parameters related to exploratory and exploitative search characteristics (McNamara et al., 2006; Elazary and Itti, 2010). These parameters include the length of memory for the prior, the distance over which sensory signals from the pellets are detected, and the relative weighting of sensory and memory terms. We allowed these parameters to vary on a multidimensional grid and analyzed goodness of fit to actual animal performance as the correlation between trial-by-trial performance of the simulated searcher and the animal (Fig. 5; see Materials and Methods). As expected, searches with long-range, noiseless sensory information lead to a perfect nearest neighbor search and do not correlate well with animal behavior (Fig. 5*B*), since rats do not have access to perfect information and need to use local sensory information or learned locations to navigate (for an examination of possible sensory cues used for this task, see Fig. 4). Similarly, searches with only a memory term also do not correlate well with actual behavior (Fig. 5*B*). Consistent with animals under different levels of uncertainty using diverse search strategies, we found that any set of a wide range of parameters applied uniformly to all animals resulted in only moderate correlation with actual behavior (Fig. 5*C*). We next allowed parameters to vary individually for each animal. While this approach will trivially result in a better fit due to the increased number of free parameters (*p* < 0.01, *n* = 12; Fig. 5*B*,*C*), we used the values of parameters obtained for these individual fits to examine the contribution of sensory and memory input to the simulated search that best matched each animal’s performance. When varying the length of memory used by the searcher we found that simulated searches across the most predictable distributions benefited from increased memory with an increase in correlation to actual animal performance when the simulated searcher had access to cumulative memory of previous searches (predictable, single trial memory: *R* = 0.12 ± 0.05; cumulative memory *R* = 0.66 ± 0.03, *p* < 0.05, *n* = 4). Searches across moderately predictable and unpredictable distributions did not show a significant increase in correlation with animal behavior with increased memory (Fig. 5*D*). Consistent with these results, the impact of shuffling prior distributions on agent performance was directly related to the predictability of the dataset (Fig. 5*E*). To quantify the impact of sensory input on these searches we combined the weighting given to sensory input with the distance from which each agent could detect a target to create a measure of sensory acuity for each simulated agent (see Materials and Methods). This measure was well correlated with increasing RE of the training set, suggesting that animals increased sensory acuity under uncertainty (*R* = 0.8469, *p* = 0.005; Fig. 5*F*, left panel). We also used the length of memory for the best match to animal behavior to create a metric for long-term memory usage (see Materials and Methods). We found a significant inverse correlation between RE and long-term memory usage (*R* = −0.7252, *p* = 0.0076; Fig. 5*F*, right panel), suggesting that as the training set became more predictable animals relied more on long-term memory. Our results are consistent with a Bayesian search where searchers adaptively shift the weightings given to various locations (and thus, their likelihood to travel to these locations) based on their relative weightings of sensory and memory terms. For example, a searcher may shift the weighting of a given location based on being rewarded there many times in the past (exploitative, memory-guided strategy) or it may shift the weighting based on sensing cues emanating from a given location (exploratory, sensory-guided strategy).

## Discussion

**Figure 6.**
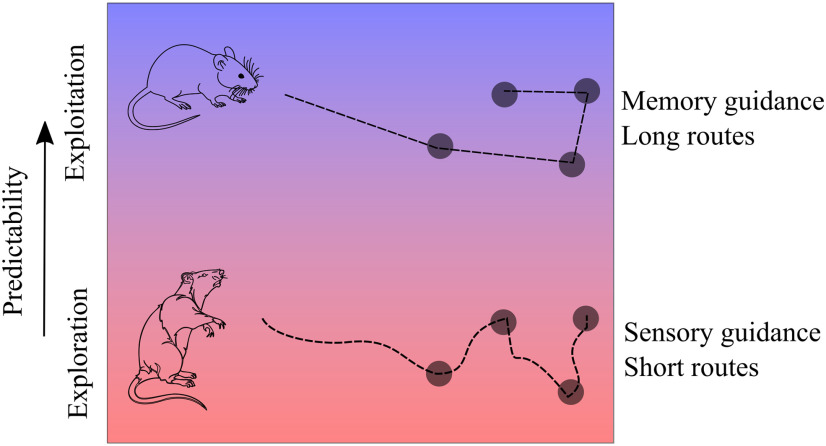
Schematic of two strategies selected to solve the probabilistic traveling salesman task. A schematic of the main results, showing that animals adaptively change the strategies used for a search depending on the level of uncertainty of the environment, here depicted as a spectrum from red (uncertain) to blue (predictable).

Animals make use of appropriate cognitive strategies and behaviors to solve the many problems they are faced with during self-guided behaviors such as foraging (Marewski and Link, 2014). It is known that when animals are introduced to new environments with multiple food locations they may continually explore and sample the different options, or they may exploit a single, most profitable option (Krebs et al., 1978). However, it is not fully understood how animals balance exploratory behaviors against exploitative behaviors (Gupta et al., 2006). Our study revealed that rodents make use of their prior knowledge of the predictability of an environment to determine the extent that they rely on sensory cues during their foraging bouts. Our results are consistent with a strategy that increases sensory acuity and reduces memory load in direct relation to the level of uncertainty in an environment (Fig. 6). This increased reliance on sensory input allows animals searching across unpredictable environments to employ an effective nearest neighbor strategy with nearly the same efficacy as animals that are operating in highly predictable environments, although due to the short-range nature of sensory cues a sensory-guided strategy fails at long distances and animals are unable to increase the efficiency of foraging trajectories over these distances (Fig. 3). Conversely, animals operating in predictable environments reduce their reliance on sensory input in favor of stereotyped and efficient searches based on long-term memory, which allows them to enhance search tours over long distances. In short, in a sensory-dominated strategy animals approach the nearest sensed pellet, while in a memory-dominated strategy animals approach the nearest remembered location, enabling more efficient, planned routes to emerge. This result is consistent with the finding that humans integrate information from different sensory modalities and dynamically give greater weight to the modality that provides the stronger, most well-defined estimate (Ernst and Banks, 2002). Taken together, these results suggest that animals assess the predictability of an environment to select appropriate strategies to allocate cognitive resources between sensory processing and memory while solving complex natural problems.

While it is difficult for animals to rapidly learn efficient paths for collecting rewards in the unpredictable environment, optimal paths in this environment are not more complex than those in predictable environments, as shown in Figure 1*D*. Indeed, animals in unpredictable environments do optimize their foraging behavior after many sessions, achieving a roughly equal ability to perform a nearest-neighbor solution to the task (Fig. 2*D*). They may learn a general understanding of where pellets have never been found (such as along the boundaries of the arena) and may focus their search to the center of the arena to maximize getting close enough to pellets to then use sensory guidance to approach the reward locations (examples in Fig. 1*B*,*H*). This suggests that while animals have a diminished, imperfect ability to rapidly learn efficient paths in unpredictable environments they are still capable of improving their foraging strategy, perhaps through a combination of coarse predictions and enhanced sensory guidance.

The differential weighting of sensory cues, specifically odor cues, is expected when the turbulent nature of odor plumes in natural environments is taken into account. Odor-guided searches are notoriously difficult due to the sparse and intermittent nature of odor plumes (Vickers, 2000). The ability of rodents to form internal representations of their environment could allow them to apply learned spatial information to dynamic environments, creating a map that would act to lessen the cognitive load required to use the complex sensory cues in odor plumes and greatly increase the effectiveness of odor-guided searches. So it follows that rodents would prefer to use a strategy that relies less on olfactory cues when instead they could navigate using the cognitive map of their familiar environment. This is in line with our results suggesting that under unpredictable conditions rats do not efficiently navigate to the next closest pellet when it is >40 cm from their current location (Fig. 3*A*). Previous research suggests that 40 cm is close to the threshold of rodents’ ability to gain a directional benefit from the sparse odor cues emanating from an odor source (Gire et al., 2016; Liu et al., 2020). This difficulty is increased when rats have been trained on unpredictable environments and are unable to construct strong expectations of pellet location. Since there is no underlying structure of where pellets can be found that animals in the unpredictable environment can learn over time, the low weighting given to the memory terms in the Bayesian model reflects animals’ discounting of information that will not be as useful as increasing their reliance on sensory cues. Animals then take advantage of the sensory cues emanating from food locations by increasing their weighting, which is in line with the results from our Bayesian model (Fig. 5). Monitoring the trajectories of the rats allowed us to also determine that rats traveled in much more efficient paths when they were navigating under conditions of high predictability. This suggests that they are able to navigate directly to where pellets are located without having to resort to behaviors indicative of searching for olfactory cues, which typically result in more circuitous search trajectories (Fig. 1*H*).

Optimizing travel paths during navigation is a notoriously difficult problem to solve, especially when one considers the complexity of the traveling salesman problem. One must determine the shortest path between multiple locations to travel efficiently and conserve the most energy or increase the rate of reward per unit time. This problem is extremely difficult to solve optimally as the complexity of the problem scales unfavorably with the number of targets that must be visited. In our task, this problem is even more complex due to the fact that animals only have probabilistic information about whether food pellets will be present at target locations. While not optimal, simplifying heuristics enable solutions to such complex optimization problems to be reached in relatively short periods of time. Nearest neighbor tours are a common strategy used to solve the traveling salesman problem (Johnson, 1990; Tsai et al., 2004). Under this strategy, the agent simply travels to the next nearest target location until all targets have been visited. While not optimal, this approach is computationally simple, resulting in rapid solutions with time to solve scaling well with task complexity. Our results suggest that animals adopt a nearest neighbor strategy to procure all of the pellets; however, the degree to which the strategy resembles a perfect nearest neighbor strategy depends on the predictability of the environment. Animals trained in a predictable environment select a strategy that highly resembles a nearest neighbor search earlier on in training (Fig. 2), which allows them to more effectively exploit pellet locations and increase efficiency (Fig. 3*A*) and speed (Fig. 3*D*) of their routes. In contrast, animals trained in unpredictable environments select a strategy that resembles a nearest neighbor search much later in training (Fig. 2*D*). These differential time courses could reflect the time necessary to train the underlying memory or sensory networks in the brain, with sensory training requiring a longer training period.

The novel, fully-automated foraging arena we designed allows for new ways to study the balance between exploration and exploitation. Using an automated, moving pellet dispenser allows for food rewards to be placed in an unlimited number of different locations throughout the foraging arena. This allows us to instantaneously change any location in the arena into a reward location. Instead of being confined to defined locations, such as fixed near a feeder, we are able to create many different distributions of where food can be found, mimicking distributions that might occur in a more naturalistic setting. By combining this automated arena with computer-generated reward distributions, we can also scale the difficulty of the task to address specific research questions. This allows us to study more complex behaviors that current experimental paradigms are not equipped to adequately explore. Through computer-aided creation of reward location sequences, our new approach also supports direct testing of algorithms that could be used to perform self-guided optimization. This task also integrates extremely well with new advances in automated behavioral tracking (Nath et al., 2019). Finally, the self-guided nature of our task allows for future studies to elucidate neural mechanisms underlying complex behaviors, such as route optimization. Since animals trained on this task are not explicitly shaped or instructed on how to best perform, we are able to study how the brain changes as animals develop solutions to complex, natural problems.

## Synthesis

Reviewing Editor: Thomas McHugh, RIKEN Center for Brain Science Decisions are customarily a result of the Reviewing Editor and the peer reviewers coming together and discussing their recommendations until a consensus is reached. When revisions are invited, a fact-based synthesis statement explaining their decision and outlining what is needed to prepare a revision will be listed below. The following reviewer(s) agreed to reveal their identity: Nathaniel Urban, Katherine Nagel.

Summary and Synthesis

In this manuscript, the authors present a new approach for examining foraging trajectories of rats in predictable and unpredictable environments. The resulting foraging trajectories are analyzed relative to optimal foraging paths and also fit to a Bayesian model in which the relative weight given to sensory and memory-derived cues can vary.

Both referees found this to be an interesting and novel study that addresses important questions about navigation and search behaviors. Following our discussion, they have agreed on several points the authors should address prior to reconsideration for publication.

There are five main points the authors should address in their revised manuscript.

1. Clarification of the statements in the introduction and results

Both reviewers agree that there are several places in the text where the data supporting a statement are not properly referenced or where a concept needs to be explained.

• The intro makes a number of statements about demands for “cognitive capacity” and energy utilization that are rather vague and suggest that the authors are considering these important factors in the strategies employed. I would recommend refocusing on optimal strategies given constraints. These tradeoffs also seem more relevant in a more naturalistic setting... the authors use a large arena, but are the paths being taken really large enough to invoke arguments about energy utilization? Perhaps, but this should be better justified.

• I am a little unclear on whether an agent can theoretically learn optimal paths for the unpredictable environment. Is the fact that paths are less optimal in the unpredictable environment a reflection of the fact that rat learning is imperfect, or that optimal paths for these environments are more complex? Similarly, is the low weight given to memory for unpredictable environments in the Bayesian model a reflection of animals limits on learning or the fact that there is no underlying structure to learn? The authors should clarify in the text what aspects of their analytical and modeling result are reflections of the structure of the environment versus animal limits/strategies in learning or taking advantage of those environments.

• p. 5 “Memory-guided exploitative strategies or sensory-guided exploratory strategies” is memory always equal to exploitation and sensory to exploration? I think of exploration as more of a noise-driven process that allows for discovery of new resources.

• p. 10 “stochastic traveling salesman problem” This needs to be explained.

2. More details on analyses and methods

• The genetic algorithm presumably can be used for “estimating” rather than “establishing” the optimal acquisition sequences - unless all possible paths are sampled. The authors need to explain here what this is, how it works, and provide some intuition for types of paths it comes up with.

• The description of sensory acuity, its computation, and which aspects of this measure are directly derived from data versus estimates based on literature needs to be better explained in both the main text, Methods, and Discussion. The claim that sensory acuity is higher in unpredictable environments is intriguing but I am not convinced by this because I was not able to understand exactly what the authors did and what evidence this is based on.

• The 10cm filter width seems large relative to the size of the arena (1m). How does the computation of RE depend on this choice of filter width?

• Fig 3 c/d: is the right measure here probability of going to the nearest pellet or latency to go to the nearest pellet? On what fraction of movements did animals not go to the nearest pellet?

3. Further analysis and examples of trajectories

The reviewers agree that the authors should provide more analyses and examples of trajectories, with Reviewer#1 noting, “I was a bit disappointed that there was not much analysis of trajectories per se but mostly analysis of sequences of visiting locations. This seems to limit the kinds of questions that can be addressed and also makes it difficult for the reader to get a general sense of the way in which these animals are performing the task. I would ask that at least some trajectories are shown and perhaps that some analysis of the features of these trajectories is performed.” Suggestions are noted below:

• One prediction that seems relevant and testable from the data is that animals employing a location-based strategy to find a food location would move more quickly than those using a sensory-based strategy. The authors should comment on this hypothesis and determine if it is supported by the data.

• In Figures 2 and 3 experimental trajectories are compared to optimal ones, as determined by a genetic algorithm. However, the authors note in the text that trajectories might be better described as nearest neighbor search. I think it would be helpful if the authors (a) showed examples of experimental trajectories under each training regime, as well as NN and optimal paths for the same environments, (b) directly compared experimental trajectories to NN search rather than to optimal search, and (c ) explain intuitively in the text how the genetic algorithm works and the types of paths it comes up with.

• p.10 “equivalent optimal path lengths” It would be helpful to show examples of these in a figure.

• p.11 “All animals favored a small subset of near-optimal acquisition sequences” The figure shown does not illustrate this very well. Please show examples of trajectories and compare to NN and “optimal” paths. Fig. 1: please show example trajectories

• p. 18 “a perfect nearest neighbor strategy depends on the predictability of the environment” Here and above it would be helpful to direct compare experimental paths to NN paths rather than to “optimal” paths.

• Fig. 1: please show example trajectories

4. Expansion and clarification of Figure 4

• The reviewers share concerns that the current Figure 4 doesn’t clearly report these data. They suggest that given that the authors have described that they mapped concentration they should report these results in a straightforward manner, even if they are somewhat messy and variable and improve the description to allow the reader to evaluate the data.

5. Additional behavioral analyses

• The results comparing behavior on predictable vs unpredictable reward locations are interesting and clearly suggest that the mice are responding to these aspects of the task. It would be useful to know if, more coarsely, animals are showing motivational differences correlated with the unpredictable reward locations. Are they running slower? spending more time sitting/grooming etc? This would help understand the cognitive vs non-cognitive aspects of the change in behavior.

Minor issues:

• p. 13 “We found that animals searching in highly predictable distributions were the most effective at navigating to nearest neighbor locations and that the advantage of predictability was strongest for large distances...” Improper figure citation. what is the data supporting this statement?

• p. 12 “Over training, animals across all predictability levels significantly increased their probability to travel to the nearest pellet during search.” Please give figure citation here.
